# Influenza A Virus Infection in Pigs Attracts Multifunctional and Cross-Reactive T Cells to the Lung

**DOI:** 10.1128/JVI.01211-16

**Published:** 2016-09-29

**Authors:** Stephanie C. Talker, Maria Stadler, Hanna C. Koinig, Kerstin H. Mair, Irene M. Rodríguez-Gómez, Robert Graage, Roland Zell, Ralf Dürrwald, Elke Starick, Timm Harder, Herbert Weissenböck, Benjamin Lamp, Sabine E. Hammer, Andrea Ladinig, Armin Saalmüller, Wilhelm Gerner

**Affiliations:** a Institute of Immunology, Department of Pathobiology, University of Veterinary Medicine, Vienna, Austria; b University Clinic for Swine, Department for Farm Animals and Veterinary Public Health, University of Veterinary Medicine, Vienna, Austria; c Department of Virology and Antiviral Therapy, Jena University Hospital, Friedrich Schiller University, Jena, Germany; d Viral Vaccines, Business Unit Animal Health, IDT Biologika GmbH, Dessau-Rosslau, Germany; e Institute of Diagnostic Virology, Friedrich-Loeffler-Institut, Federal Research Institute for Animal Health, Greifswald-Insel Riems, Germany; f Institute of Pathology and Forensic Veterinary Medicine, Department of Pathobiology, University of Veterinary Medicine, Vienna, Austria; g Institute of Virology, Department of Pathobiology, University of Veterinary Medicine, Vienna, Austria; St. Jude Children's Research Hospital

## Abstract

Pigs are natural hosts for influenza A viruses and play a critical role in influenza epidemiology. However, little is known about their influenza-evoked T-cell response. We performed a thorough analysis of both the local and systemic T-cell response in influenza virus-infected pigs, addressing kinetics and phenotype as well as multifunctionality (gamma interferon [IFN-γ], tumor necrosis factor alpha [TNF-α], and interleukin-2 [IL-2]) and cross-reactivity. A total of 31 pigs were intratracheally infected with an H1N2 swine influenza A virus (FLUAVsw) and consecutively euthanized. Lungs, tracheobronchial lymph nodes, and blood were sampled during the first 15 days postinfection (p.i.) and at 6 weeks p.i. *Ex vivo* flow cytometry of lung lymphocytes revealed an increase in proliferating (Ki-67^+^) CD8^+^ T cells with an early effector phenotype (perforin^+^ CD27^+^) at day 6 p.i. Low frequencies of influenza virus-specific IFN-γ-producing CD4^+^ and CD8^+^ T cells could be detected in the lung as early as 4 days p.i. On consecutive days, influenza virus-specific CD4^+^ and CD8^+^ T cells produced mainly IFN-γ and/or TNF-α, reaching peak frequencies around day 9 p.i., which were up to 30-fold higher in the lung than in tracheobronchial lymph nodes or blood. At 6 weeks p.i., CD4^+^ and CD8^+^ memory T cells had accumulated in lung tissue. These cells showed diverse cytokine profiles and *in vitro* reactivity against heterologous influenza virus strains, all of which supports their potential to combat heterologous influenza virus infections in pigs.

**IMPORTANCE** Pigs not only are a suitable large-animal model for human influenza virus infection and vaccine development but also play a central role in the emergence of new pandemic strains. Although promising candidate universal vaccines are tested in pigs and local T cells are the major correlate of heterologous control, detailed and targeted analyses of T-cell responses at the site of infection are scarce. With the present study, we provide the first detailed characterization of magnitude, kinetics, and phenotype of specific T cells recruited to the lungs of influenza virus-infected pigs, and we could demonstrate multifunctionality, cross-reactivity, and memory formation of these cells. This, and ensuing work in the pig, will strengthen the position of this species as a large-animal model for human influenza virus infection and will immediately benefit vaccine development for improved control of influenza virus infections in pigs.

## INTRODUCTION

In 2016, almost 100 years after the devastating 1918 influenza pandemic in humans, influenza A viruses remain a challenge for vaccine development. Antigenic drift and reassortment of influenza virus genomes enable evasion from serological herd immunity, resulting in annual epidemics and unpredictable pandemic outbreaks (1). Reassortment often occurs in pigs, which are susceptible to both avian and human-adapted influenza viruses (2), and these animals have therefore been suggested as mixing vessels, providing ideal conditions for the production of new pandemic strains (3, 4). Bidirectional influenza virus transmissions between humans and pigs are known to occur frequently (5–9). The pandemic outbreak of “swine flu” in 2009 demonstrated how easily reassorted strains of pig origin can “jump” to a naive human population (10) and led to calls for increased surveillance and improved control of influenza in pigs (11–13).

Current influenza vaccines for both pigs and humans elicit primarily strain-specific humoral immunity, failing to protect against strains carrying drift variants or reassorted genome segments of hemagglutinin (HA). In the quest to develop broadly protective vaccines, T cells have increasingly gained attention, as they are able to recognize internal epitopes that are highly conserved across influenza virus subtypes (14). The important role of T cells in the clearance of influenza in mice (15, 16) and their cross-reactive potential (17, 18) have long been known. More recent mouse studies have provided evidence that memory T cells in the lung are key to protect against influenza virus infection (19–22). Evidence for a protective role of T cells also comes from nonhuman primate models (23, 24), which more closely approximate human infection. In humans, preexisting influenza virus-reactive T cells and the rapid onset of influenza virus-specific T-cell responses, as measured in blood, could be correlated with reduced symptom scores and rapid recovery from infection, respectively (25–27). Local lung responses are difficult to assess in humans, but influenza virus-reactive T cells with a tissue-resident memory phenotype could be detected in human lungs obtained by lobectomy (28, 29) and from organ donors (30).

Despite the serious zoonotic threat posed by influenza virus-infected pigs and their suitability as large-animal models for human vaccine development (31), in-depth data on porcine T-cell immunity to influenza virus are scarce. Many studies support the involvement of T cells in porcine influenza virus infection (32–41), but only recently we reported on the first comprehensive study of T-cell kinetics, phenotype, and quality (42). We were able to demonstrate influenza virus specificity, multifunctionality, and memory responses of blood-derived CD4^+^ and CD8^+^ T cells in pigs. These longitudinal data from peripheral blood clearly supported an important role of T cells in the porcine immune response against influenza virus infection, though we emphasized the urgent need for T-cell analyses at the site of infection. With the present study, we provide the first thorough characterization of the local T-cell response in the porcine influenza virus-infected lung, addressing kinetics and phenotype, as well as cytokine profile, memory formation, and cross-reactivity.

## MATERIALS AND METHODS

### Animal infection experiments.

**FIG 1:**
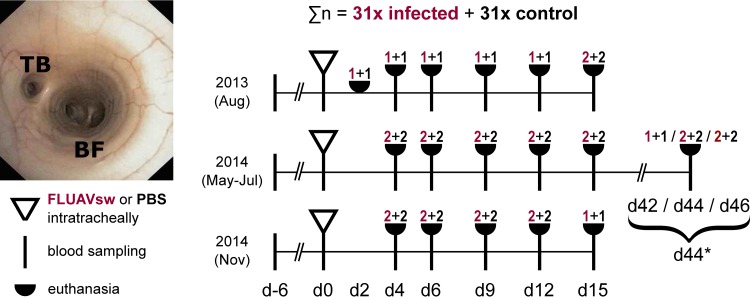
Animal infection experiments. A total of 62 10-week-old pigs were used in three consecutive animal infection experiments. The overall experiment was split into three separate animal infection experiments, which were performed sequentially, to allow for efficient working practices. In each experiment, the animals were given an adaptation phase of 6 days, before FLUAVsw (infected, *n* = 31) or PBS (control, *n* = 31) was applied intratracheally. Application was performed under visual control of a bronchoscope. The photograph shows the tracheal bronchus (TB) and tracheal bifurcation (BF) as seen through the bronchoscope when positioned for application. For tissue collection, animals were euthanized on the indicated days postinfection. Blood samples were taken from both euthanized and surviving animals. The number of euthanized animals (infected plus control) is denoted separately for each study day and makes a total of 5 infected and 5 control animals per time point (with the exception of day 2 p.i., when only two pigs were euthanized). *, the late time points (days 42, 44, and 46) served to address memory responses of T cells; results obtained on these study days were combined and are referred to as “day 44 p.i.” throughout this article.

In three consecutive animal infection experiments, a total of 31 10-week-old pigs were administered swine influenza A virus (H1N2 FLUAVsw) intratracheally. Another 31 pigs underwent the same procedure but received phosphate-buffered saline (PBS) instead (Fig. 1). Pigs ([Landrace × Large White] × Pietrain) were obtained from a conventional breeding farm in the federal state of Lower Austria. Breeding sows on this farm had repeatedly tested negative for anti-influenza A virus nucleoprotein antibodies (ID Screen Influenza A Antibody Competition Multispecies enzyme-linked immunosorbent assay [ELISA]; IDvet, Grabels, France) as well as antibodies against porcine reproductive and respiratory syndrome virus (PRRSV) (tested by PRRS X3 ELISA; Idexx, Ludwigsburg, Germany). The study pigs also tested negative for anti-influenza A virus nucleoprotein antibodies prior to experimental infection. At 3 weeks of age, the study pigs were vaccinated, in accordance with routine veterinary practice in Austria, against *Mycoplasma hyopneumoniae* (MycoFLEX; Boehringer Ingelheim, Ingelheim, Germany) and porcine circovirus type 2 (CircoFLEX; Boehringer Ingelheim). The animal infection experiments took place under biosafety level 2 containment at the University of Veterinary Medicine, Vienna, Austria. Transfer of animals and random allocation to infection and control groups was done 1 week prior to FLUAVsw/PBS application (day −6). On the day of infection, the pigs were anesthetized (Narketan [ketamine hydrochloride] at 10 mg/kg body weight [BW] intramuscularly [i.m.] [Vetoquinol, Lure, France] and Stresnil [azaperone] at 1.2 mg/kg BW i.m. [Janssen Pharmaceutical, Beerse, Belgium]) and administered either 15 ml of virus suspension (FLUAVsw [A/swine/Kitzen/IDT6142/2007; H1N2]; 10^7.25^ 50% tissue culture infective doses [TCID_50_]/ml) or 15 ml of PBS (without Ca^2+^ and Mg^2+^; PAN Biotech, Aidenbach, Germany) intratracheally. A bronchoscope was used to ensure standardized application proximal to the tracheal bronchus (Fig. 1). One infected pig and one control pig were euthanized at day 2 postinfection (p.i.). Five infected pigs and five control pigs were euthanized consecutively on days 4, 6, 9, 12, 15, and 42 to 46 p.i. (days 42 to 46 p.i. are referred to here as “day 44”) (Fig. 1). For euthanasia, pigs were anesthetized (Narketan and Stresnil, both i.m.), and T61 (embutramide, mebezonium iodide, and tetracaine hydrochloride at 1 ml/10 kg BW; MSD, Whitehouse Station, NJ) was administered intracardially. Swine leukocyte antigen (SLA) haplotyping of pigs was performed as previously described (43). The low-resolution SLA haplotypes of all infected pigs are given in Table 1. The animal infection experiment was approved by the institutional ethics committee and the national authority according to section 26 of the Law for Animal Experiments, Tierversuchsgesetz 2012 – TVG 2012 (reference number BMWF-68.205/0103-II/3b/2013).

**TABLE 1:** SLA haplotypes of infected pigs

Pig	Trial	Day of euthanasia	SLA class I				SLA class II			
			Haplotype	SLA allele specificity			Haplotype	SLA allele specificity		
				SLA-1	SLA-3	SLA-2		DRB1	DQB1	DQA
1	1	2	Lr-28.0	09XX, 15XX	07XX	05XX	Lr-0.23	10XX	06XX	01XX
			Lr-24.0	Blank	04XX	06XX	Lr-0.19a	04XX	07XX	03XX
2	1	4	Lr-01.0	01XX	01XX	01XX	Lr-0.15b	04XX	02XX	02XX
			Lr-67.0	15XX	05XX	10XX	Lr-0.20	06XX	03XX	01XX
3	2	4	Lr-04.0	04XX	04XX	04XX	Lr-0.01	04XX	08XX	03XX
			Lr-43.0	11XX	04XX	04XX	Lr-0.29	Blank	09XX	04XX
4	2	4	Lr-28.0	09XX, 15XX	07XX	05XX	Lr-0.23	10XX	06XX	01XX
			Lr-24.0	Blank	04XX	06XX	Lr-0.20	06XX	03XX	01XX
5	3	4	Lr-04.0	04XX	04XX	04XX	Lr-0.23	10XX	06XX	01XX
			Lr-28.0	09XX, 15XX	07XX	05XX	Lr-0.11	09XX	04XX	03XX
6	3	4	Lr-04.0	04XX	04XX	04XX	Lr-0.23	10XX	06XX	01XX
			Lr-28.0	09XX, 15XX	07XX	05XX	Lr-0.11	09XX	04XX	03XX
7	1	6	Lr-24.0	Blank	04XX	06XX	Lr-0.15b	04XX	02XX	02XX
			Lr-05.0	04XX	05XX	w08XX	Lr-0.19a	04XX	07XX	03XX
8	2	6	Lr-32.0	07XX	04XX	02XX	Lr-0.23	10XX	06XX	01XX
			Lr-32.0	07XX	04XX	02XX	Lr-0.15b	04XX	02XX	02XX
9	2	6	Lr-43.0	11XX	04XX	04XX	Lr-0.08b	08XX	0203	02XX
			Lr-07.0	08XX	07XX	05XX	Lr-0.11	09XX	04XX	03XX
10	3	6	Lr-26.0	08XX	05XX	10XX	Lr-0.23	10XX	06XX	01XX
			Lr-28.0	09XX, 15XX	07XX	05XX	Lr-0.20	06XX	03XX	01XX
11	3	6	Lr-24.0	Blank	04XX	06XX	Lr-0.11	09XX	04XX	03XX
			Lr-43.0	11XX	04XX	04XX	Lr-0.23	10XX	06XX	01XX
12	1	9	Lr-28.0	09XX, 15XX	07XX	05XX	Lr-0.23	10XX	06XX	01XX
			Lr-59.0	11XX	05XX	jh02	Lr-0.23	10XX	06XX	01XX
13	2	9	Lr-28.0	09XX, 15XX	07XX	05XX	Lr-0.23	10XX	06XX	01XX
			Lr-04.0	04XX	04XX	04XX	Lr-0.26	11XX	04XX	02XX
14	2	9	Lr-05.0	04XX	05XX	w08XX	Lr-0.11	09XX	04XX	03XX
			Lr-43.0	11XX	04XX	04XX	Lr-0.20	06XX	03XX	01XX
15	3	9	Lr-66.0	15XX	04XX	04XX	Lr-0.11	09XX	04XX	03XX
			Lr-43.0	11XX	04XX	04XX	Lr-0.15b	04XX	02XX	02XX
16	3	9	Lr-28.0	09XX, 15XX	07XX	05XX	Lr-0.23	10XX	06XX	01XX
			Lr-24.0	Blank	04XX	06XX	Lr-0.6	05XX	08XX	01XX
17	1	12	Lr-24.0	Blank	04XX	06XX	Lr-0.19a	04XX	07XX	03XX
			Lr-24.0	Blank	04XX	06XX	Lr-0.23	10XX	06XX	01XX
18	2	12	Lr-04.0	04XX	04XX	04XX	Lr-0.23	10XX	06XX	01XX
			Lr-28.0	09XX, 15XX	07XX	05XX	Lr-0.23	10XX	06XX	01XX
19	2	12	Lr-05.0	04XX	05XX	w08XX	Lr-0.11	09XX	04XX	03XX
			Lr-43.0	11XX	04XX	04XX	Lr-0.15b	04XX	02XX	02XX
20	3	12	Lr-29.0	Blank (08XX?)	05XX	w09XX	Lr-0.23	10XX	06XX	01XX
			Lr-35.0	12XX, 13XX	05XX	10XX	Lr-0.23	10XX	06XX	01XX
21	3	12	Lr-01.0	01XX	01XX	01XX	Lr-0.15b	04XX	02XX	02XX
			Lr-39.0	Blank	05XX	10XX	Lr-0.23	10XX	06XX	01XX
22	1	15	Lr-01.0	01XX	01XX	01XX	Lr-0.15b	04XX	02XX	02XX
			Lr-67.0	15XX	05XX	10XX	Lr-0.20	06XX	03XX	01XX
23	1	15	Lr-26.0	08XX	05XX	10XX	Lr-0.23	10XX	06XX	01XX
			Lr-28.0	09XX, 15XX	07XX	05XX	Lr-0.20	06XX	03XX	01XX
24	2	15	Lr-28.0	09XX, 15XX	07XX	05XX	Lr-0.23	10XX	06XX	01XX
			Lr-35.0	12XX, 13XX	05XX	10XX	Lr-0.23	10XX	06XX	01XX
25	2	15	Lr-04.0	04XX	04XX	04XX	Lr-0.08b	08XX	02XX	02XX
			Lr-34.0	Blank (15XX, 11XX)	04XX	05XX	Lr-0.11	09XX	04XX	03XX
26	3	15	Lr-28.0	09XX, 15XX	07XX	05XX	Lr-0.23	10XX	06XX	01XX
			Lr-43.0	11XX	04XX	04XX	Lr-0.11	09XX	04XX	03XX
27	2	42	Lr-07.0	08XX	07XX	05XX	Lr-0.23	10XX	06XX	01XX
			Lr-28.0	09XX, 15XX	07XX	05XX	Lr-0.08b	08XX	0203	02XX
28	2	44	Lr-66.0	15XX	04XX	04XX	Lr-0.26	11XX	04XX	02XX
			Lr-25.0	11XX	03XX	07XX	Lr-0.19a	04XX	07XX	03XX
29	2	44	Lr-66.0	15XX	04XX	04XX	Lr-0.11	09XX	04XX	03XX
			Lr-35.0	12XX, 13XX	05XX	10XX	Lr-0.26	11XX	04XX	02XX
30	2	46	Lr-04.0	04XX	04XX	04XX	Lr-0.11	09XX	04XX	03XX
			Lr-25.0	11XX	03XX	07XX	Lr-0.15b	04XX	02XX	02XX
31	2	46	Lr-01.0	01XX	01XX	01XX	Lr-0.14	09XX	08XX	03XX
			Lr-43.0	11XX	04XX	04XX	Lr-0.24	07XX	02XX	02XX

### Influenza A virus and recombinant nucleoprotein.

Influenza A/swine/Kitzen/IDT6142/2007 (H1N2) was used for infection and homologous restimulation experiments. It belongs to the human-like H1N2 swine influenza virus lineage (Table 2) and harbors the H1 of human seasonal, pre-2009 influenza viruses. Further details regarding this strain have been described by Talker et al. (42).

**TABLE 2:** Genetic constellation of the four influenza A viruses used in this study

Isolate	Protein origin^*a*^								Accession no.
	PB2	PB1	PA	HA	NP	NA	M1	NS1	
A/swine/Kitzen/IDT6142/2007 (H1N2)	Av	Av	Av	Hu H1	Av	Hu N2	Av	Av	KX433131–KX433136^*b*^
									GQ161145^*b*^, GQ161146
A/swine/Germany/R1738/2010 (H1avN1)	Av	Av	Av	Av H1	Av	Av N1	Av	Av	EPI_ISL_222129^*c*^
A/swine/Germany/AR1190/2014 (H1avN2)	Av	Av	Av	Av H1	Av	Hu N2	Av	Av	EPI_ISL_222085^*c*^
A/swine/Germany/R655/2012 (H3N2)	Av	Av	Av	Hu H3	Av	Hu N2	Av	Av	EPI_ISL_133484^*c*^

a Av, avian origin; Hu, human seasonal origin.

b GenBank accession number.

c GISAID EpiFlu database accession number.

For heterologous *in vitro* restimulation experiments, the following influenza virus isolates were used: A/swine/Germany/R1738/2010 (H1N1), A/swine/Germany/AR1190/2014 (H1N2), and A/swine/Germany/R655/2012 (H3N2). The HAs of R1738/10 and AR1190/14 belong to the avian-derived European FLUAVsw lineages. R655/12 belongs to HA subtype H3. The neuraminidase (NA) N1 of R1738/10 is of avian origin, while the N2s of the other three viruses belong to different human seasonal strains of H3N2 lineages (Table 2). These isolates were obtained from nasal swabs of pigs with overt respiratory disease and were passaged twice in MDCK cells as previously described (44). Virus titers were determined in the same cell type using trypsin-supplemented, serum-free medium. Titer calculation with the Kaerber formula was based on cytopathic effects and staining of viral nucleocapsid antigen using the monoclonal antibody ATCC Hb-65. Full-length genome sequences were available from public databases or have previously been established by Sanger sequencing as described by Harder et al. (44). Sequence accession numbers are shown in Table 2. Lineage identity (avian versus human origin) was assigned according to the results of extensive homology searches using the Influenza Research Database (www.fludb.org). Pairwise comparison of identity measures of deduced protein sequences from all eight genome segments were obtained using GeneDoc software (www.nrbsc.org/gfx/genedoc/gdfeedb.htm).

Recombinant influenza A virus nucleoprotein (rNP) was purchased from Sino Biological Inc. (Beijing, China) (DNA sequence derived from A/Anhui/1-BALF_RG6/2013, H7N9; accession number: AHZ59787.1).

### Clinical and pathological examination.

**FIG 2:**
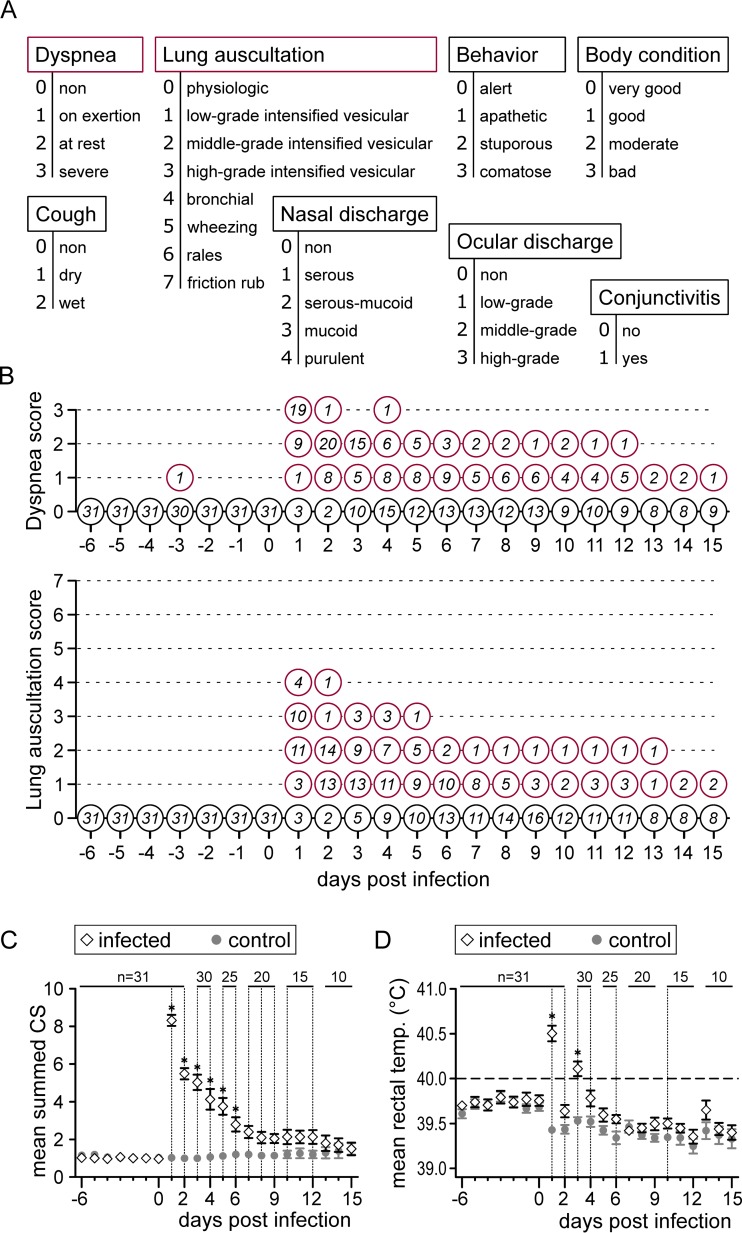
Clinical findings. Clinical examinations were performed daily from 6 days prior to infection until 15 days p.i. (A) A scoring system was used to evaluate a total of eight clinical parameters. (B) For the two most severe parameters (dyspnea and lung auscultation), the number of animals per score is given over the course of the infection. (C and D) Mean values of summed clinical scores (CS) (C) and rectal temperatures (D) for the infected group (diamonds) and the control group (gray dots). For each day, group means were compared by independent *t* tests (vertical dashed lines, *P* < 0.05; asterisks, *P* < 0.001). Error bars show the standard error of the mean.

**FIG 3:**
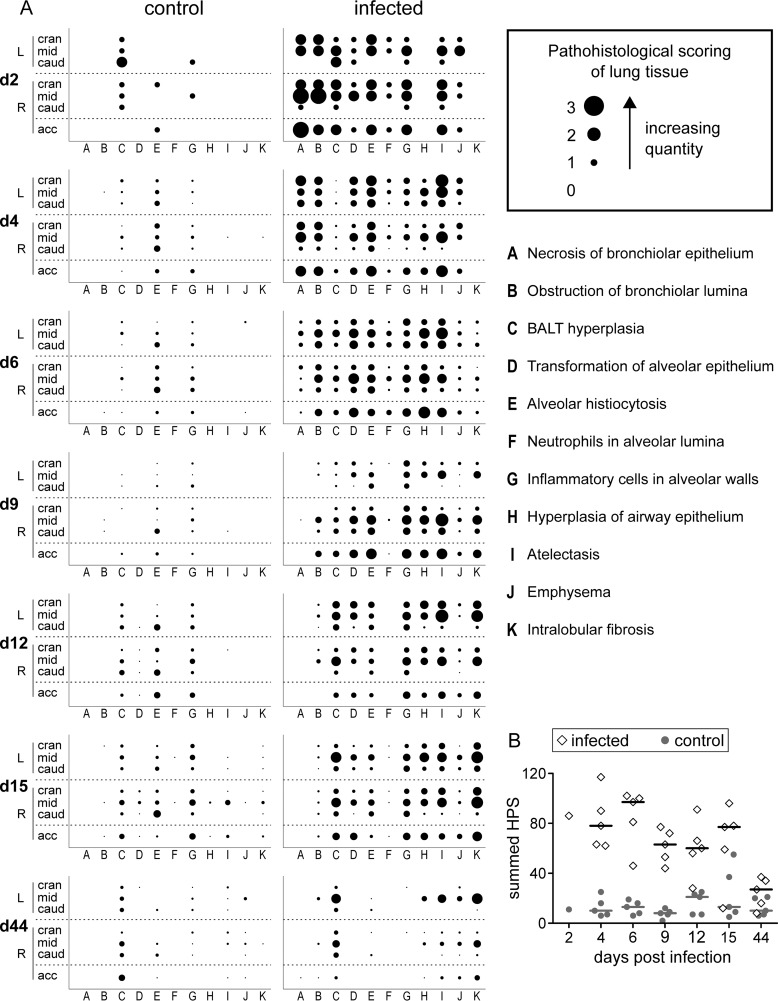
Histopathology of lung tissue. (A) Paraffin sections of all seven lung lobes (left cranial, middle, and caudal and right cranial, middle, caudal, and accessory) from PBS-treated control pigs and FLUAVsw-infected pigs were stained with hematoxylin and eosin and examined for the presence and quantity of parameters A to K (defined in the figure). The quantity and presence of each parameter were recorded as a score (0 [absent] to 3 [high grade]). Dot sizes represent the score for one individual animal (day 2) or the mean score for five animals (days 4 to 44). (B) Histopathology scores (HPS) were summed for each individual animal (infected, diamonds; control, gray dots). Horizontal bars indicate the median value for five animals.

Clinical examinations, including the measurement of rectal temperatures, were carried out on a daily basis. A scoring system was used to document respiratory signs, behavior, and body condition (Fig. 2A). At necropsy, gross lung lesions were documented, and samples from all seven lung lobes were fixed in 10% neutral buffered formalin for histological investigation. Paraffin sections (3 μm) were stained with hematoxylin and eosin, and 11 different pathological parameters were quantified using scores from 0 (absent) to 3 (high grade) (Fig. 3A).

### Sample collection and isolation of lymphocytes.

Lung tissue, tracheobronchial lymph nodes (TBLN), mesenteric lymph nodes (MesLN), and bronchoalveolar lavage fluid (BALF) were collected at euthanasia. Blood samples were taken from the jugular veins of euthanized as well as surviving animals. Serum was frozen at minus 20°C for use in serum neutralization assays. Peripheral blood mononuclear cells (PBMC) were isolated from heparinized blood by density gradient centrifugation (Pancoll human; density, 1.077 g/ml; PAN Biotech) as described elsewhere (45). Tracheobronchial lymph nodes and mesenteric lymph nodes were processed for isolation of lymphocytes as previously described (46). For the isolation of lymphocytes from lung tissue, a sample of tissue (approximately 4 by 3 by 2 cm) from the center of the right cranial lobe was snipped into small pieces (approximately 3 by 3 by 3 mm), rinsed with PBS to reduce contamination from blood, and shake incubated at 37°C for 1 h in 25 ml of digestion medium (RPMI 1640 with stable glutamine supplemented with 2% fetal calf serum [FCS], 100 IU/ml penicillin, and 0.1 mg/ml streptomycin [all from PAN Biotech], 20 mM HEPES [Sigma-Aldrich, Schnelldorf, Germany], 25 U/ml DNase type 1 [Invitrogen, Waltham, MA], and 300 U/ml collagenase type 1 [Invitrogen]). Digested tissue was sieved through cotton wool, spun down (350 × *g*, 10 min, room temperature), resuspended in 30 ml PBS, and layered onto 15 ml of lymphocyte separation medium (Pancoll human; PAN Biotech). After centrifugation (920 × *g*, 30 min, room temperature) and collection of the interphase, cells were washed (350 × *g*, 10 min, 4°C) twice with PBS and once with wash medium (RPMI 1640 with stable glutamine supplemented with 5% FCS, 100 IU/ml penicillin, and 0.1 mg/ml streptomycin). Cells seeded for *in vitro* restimulation were resuspended in cell culture medium (RPMI 1640 with stable glutamine supplemented with 10% FCS, 100 IU/ml penicillin, and 0.1 mg/ml streptomycin). Bronchoalveolar lavage fluid was obtained postmortem. An incision was made into the trachea, a tracheal tube was inserted, and the lung was flushed with 100 ml of PBS. BALF was spun down (350 × *g*, 10 min, 4°C), and supernatant was frozen at minus 80°C for detection of viral RNA in subsequent quantitative reverse transcription-PCR (qRT-PCR) experiments.

### *Ex vivo* determination of phenotype and Ki-67 expression.

For *ex vivo* flow cytometric (FCM) analyses, freshly isolated cells from lung and tracheobronchial lymph nodes were suspended in PBS (without Ca^2+^ and Mg^2+^; PAN Biotech) supplemented with 10% (vol/vol) porcine plasma (in-house preparation) and adjusted to 1.5 × 10^6^ cells per sample. Staining was performed in 96-well round-bottom plates (with incubations for 20 min at 4°C). Antibodies and secondary reagents are listed in Table 3. A Live/Dead Near-IR stain kit (Invitrogen) was used for the discrimination of dead cells. For fixation and permeabilization, the Foxp3 staining buffer set (eBioscience, San Diego, CA) was used according to the manufacturer's instructions.

**TABLE 3:** Antibody panels

Purpose	Antigen	Clone	Isotype	Fluorochrome^*a*^	Labeling strategy	Source of primary antibody
*Ex vivo* Ki-67 expression	CD3	BB23-8E6-8C8	IgG2a	PE-Cy7	Directly conjugated	BD Biosciences
	CD4	74-12-4	IgG2b	PerCP-Cy5.5	Directly conjugated	BD Biosciences
	CD8α	11/295/33	IgG2a	Alexa 647	Directly conjugated^*b*^	In-house
	CD8β	PG164A	IgG2a	Alexa 488	Zenon labeling kit^*c*^	VMRD
	CD27	b30c7	IgG1	BV605	Biotin-streptavidin^*d*^	In-house
	Perforin	δ-G9	IgG2b	PE	Directly conjugated	eBioscience
	Ki-67	B56	IgG1	BV421	Directly conjugated	BD Biosciences
Triple cytokine staining of freshly isolated cells	CD4	74-12-4	IgG2b	PerCP-Cy5.5	Directly conjugated	BD Biosciences
	CD8α	76-2-11	IgG2a	PE-Cy7	Secondary antibody^*e*^	BD Biosciences
	CD8β	PG164A	IgG2a	Alexa 488	Zenon labeling kit^*c*^	VMRD
	CD27	b30c7	IgG1	BV421	Biotin-streptavidin^*f*^	In-house
	IFN-γ	P2G10	IgG1	PE	Directly conjugated	BD Biosciences
	TNF-α	MAb11	IgG1	BV605	Directly conjugated	BioLegend
	IL-2	A150D3F1	IgG2a	APC	Directly conjugated^*g*^	Invitrogen
Triple cytokine staining of defrosted cells	CD4	74-12-4	IgG2b	PerCP-Cy5.5	Directly conjugated	BD Biosciences
	CD8α	76-2-11	IgG2a	PE-Cy7	Secondary antibody^*e*^	BD Biosciences
	CD8β	PPT23	IgG1	BV421	Biotin-streptavidin^*f*^	In-house
	IFN-γ	P2G10	IgG1	PE	Directly conjugated	BD Biosciences
	TNF-α	MAb11	IgG1	BV605	Directly conjugated	BioLegend
	IL-2	A150D3F1	IgG2a	APC	Directly conjugated^*g*^	Invitrogen

a PE, phycoerythrin; PerCP, peridinin chlorophyll protein; APC, allophycocyanin.

b Alexa Fluor 647 protein labeling kit; Invitrogen.

c Zenon Alexa Fluor 488 mouse IgG2a labeling kit; Invitrogen.

d Brilliant violet 605 streptavidin; BioLegend.

e Goat anti-mouse IgG2a-PE-Cy7; SouthernBiotech.

f Brilliant violet 421 streptavidin; BioLegend.

g Lightning-Link APC conjugation kit; Innova Biosciences.

### ELISpot assays for gamma interferon (IFN-γ) production.

Enzyme-linked immunosorbent spot (ELISpot) assays were performed as previously described (42). For *in vitro* restimulation, 3 × 10^5^ cells, freshly isolated from blood (PBMC), lung, tracheobronchial lymph nodes, and mesenteric lymph nodes, were incubated with FLUAVsw (infection strain; multiplicity of infection [MOI] = 0.1) for 24 h at 37°C. Mesenteric lymph nodes (MesLN) were analyzed as control tissues. Medium and mock incubations served as additional negative controls, as did cells isolated from noninfected control animals. For restimulation experiments with heterologous virus and nucleoprotein, PBMC and lung cells were defrosted and incubated for 24 h with virus (MOI = 0.3) or nucleoprotein (1 μg/ml), or were mock infected, at 37°C.

### Intracellular cytokine staining (ICS).

To analyze production of IFN-γ, tumor necrosis factor alpha (TNF-α), and interleukin-2 (IL-2) by FLUAVsw-specific CD4^+^ and CD8^+^ T cells, freshly isolated cells from blood, lung, and tracheobronchial lymph nodes were restimulated *in vitro* with FLUAVsw (infection strain, MOI = 0.1) or mock treated for 18 h at 37°C. For restimulation experiments including heterologous viruses and nucleoprotein, PBMC and lung cells were defrosted and rested for 8 h before virus (MOI = 0.3), or nucleoprotein (1 μg/ml) was added (incubation for 18 h at 37°C) or mock treatment applied. As an additional control, defrosted PBMC were also stimulated with a live attenuated PRRSV genotype 1 strain (ReproCyc PRRS EU; Boehringer Ingelheim Limited, Bracknell, United Kingdom) (MOI = 0.2). Cells were cultured in round-bottom 96-well plates at 5 × 10^5^ cells in a volume of 200 μl per well. Brefeldin A (BD GolgiPlug; BD Biosciences, San Jose, CA) was added 4 h prior to harvesting of cells at a final concentration of 1 μg/ml. For FCM staining, cells were harvested and rebuffered with PBS (without Ca^2+^ and Mg^2+^) containing 3% FCS. Staining was performed in round-bottom 96-well plates. Antibodies and secondary reagents are listed in Table 3. Free binding sites of secondary antibodies were blocked with whole mouse IgG molecules (2 μg per sample; Jackson ImmunoResearch, West Grove, PA). A Live/Dead Near-IR stain kit (Invitrogen) or Fixable Viability Dye eFluor780 (eBioscience) was used to discriminate dead cells. BD Cytofix/Cytoperm and BD Perm/Wash (both from BD Biosciences) were used according to the manufacturer's instructions.

### FCM analysis.

A FACSCanto II flow cytometer (BD Biosciences) equipped with three lasers (405, 488, and 633 nm) was used for analysis. Single-stain samples were included for automated calculation of compensation. From the *ex vivo* stainings, 2 × 10^5^ lymphocytes were recorded using the high-throughput sampler of the flow cytometer. Cells subjected to overnight restimulation and intracellular cytokine staining were analyzed in FCM tubes, yielding between 5 × 10^5^ and 1 × 10^6^ recorded lymphocytes per sample. Doublet discrimination and dead-cell exclusion were performed as previously described (42). Data were processed by FACSDiva software (version 6.1.3; BD Biosciences) and transferred to Microsoft Excel (Office 2010; Microsoft, Redmond, WA) for further calculations.

### Serum neutralization test.

Infection strain neutralizing antibody titers in defrosted sera were analyzed as described elsewhere (47).

### qRT-PCR.

Viral loads in BALF were assessed following a TaqMan one-step quantitative reverse transcription-PCR (qRT-PCR) assay in a 7300 real-time PCR System (Applied Biosystems, Foster City, CA). RNA was extracted from BALF with the QIAamp viral RNA minikit (Qiagen, Valencia, CA), obtaining 60 μl of eluted RNA. M fragment amplification was carried out using two primers (FLUAM-1F [nucleotides {nt} 169 to 191; 5′-AAGACCAATCCTGTCACCTCTGA-3′] and FLUAM-1R [nt 263 to 242; 5′-CAAAGCGTCTACGCTGCAGTCC-3′]) yielding an RT-PCR amplicon of 94 bp, and quantification was facilitated by a specific probe (FLUAM-1P [nt 209 to 228; 5′-FAM-TTTGTGTTCACGCTCACCGT-TAMRA-3′]) (48). SuperScript III one-step qRT-PCR kit reagents (Invitrogen) and oligonucleotide concentrations were used according to the manufacturer's instructions, applying 2.5 μl of eluted RNA to a total reaction volume of 25 μl. The amplification conditions were as follows: reverse transcription at 50°C for 15 min, initial denaturation reaction at 95°C for 2 min, and 45 PCR cycles of 95°C for 15 s and 60°C for 30 s. A recombinant plasmid encoding the M fragment sequence was purified using the QIAprep spin kit (Qiagen) and spectrophotometrically quantified (Qubit; Invitrogen). The copy numbers of recombinant plasmids were calculated following the formula *N* (molecules per microliter) = (*C* [DNA, μg/μl]/*K* [fragment size in bp]) × 185.5 × 10^13^ (factors derived from DNA weight, volume, and the Avogadro constant). Serial 10-fold dilutions of the plasmid were made using 1 × 10^1^ to 1 × 10^7^ copies of the recombinant plasmid with the M gene fragment per reaction to generate the standard curves. The genome equivalent copies (GEC) from the collected BALF samples were determined based on a standard curve, taking their volumes into account. Since the efficiencies of RNA purification and the reverse transcription reaction were not determined, GEC numbers do not exactly reflect the number of viral RNA molecules in samples.

### Statistics and preparation of figures.

Statistical analyses were performed using SPSS (SPSS Inc., IBM, Chicago, IL) version 22. Independent sample *t* tests were used to compare mean summed clinical scores and mean rectal temperatures between the infected group and the control group. Figures were prepared using GraphPad Prism version 5.04 for Windows (GraphPad Software, San Diego, CA) and Inkscape (www.inkscape.org).

## RESULTS

### Clinical signs, histopathology, and viral load.

All 31 FLUAVsw-inoculated pigs developed clinical signs indicative of lower respiratory tract infection, with the most prominent signs being dyspnea, coughing, and abnormal breathing sounds upon lung auscultation (Fig. 2B and data not shown). These respiratory signs were accompanied by pyrexia (rectal temperature of >40°C [physiological range for growing pigs, 38.7 to 39.3°C]), and lethargy. Inflammatory signs of the upper respiratory tract (nasal/ocular discharge and conjunctivitis) were absent in almost all animals (data not shown). Mean summed clinical scores (CS) were highest on day 1 p.i., and within 1 week they gradually declined to levels similar to those of the PBS-treated control animals (Fig. 2C). Rectal temperatures showed a biphasic pattern, with an initial peak on day 1 p.i. followed by a second peak on day 3 p.i. (Fig. 2D). At necropsy, gross lesions were restricted to the lungs of infected animals (1 to 10% of total lung surface). During the acute phase of infection (day 4 to day 15 p.i.), gross lung lesions included tissue consolidation, atelectasis, and emphysema. At 6 weeks p.i. (days 42, 44, and 46), fibrotic alterations were observed in the lungs of four out of five animals (data not shown).

These findings were confirmed microscopically, where a scoring system was used to quantify 11 different alterations. An overview of the 11 different histological alterations, their quantity, and their location with respect to the lung lobes is given in Fig. 3A. The most prominent findings were, among others, necrosis of bronchiolar epithelium (days 2 and 4 p.i.), obstruction of bronchiolar lumina, alveolar histiocytosis, inflammatory cells in alveolar walls, and atelectasis. Intralobular fibrosis, an indicator of tissue repair, was observed from day 6 to 9 p.i. onwards. Generally, accessory, cranial, and middle lung lobes were affected more severely than caudal lobes. By the end of the study, on day 44 p.i., lungs of infected animals showed residual hyperplasia of bronchus-associated lymphoid tissue (BALT), atelectasis, and intralobular fibrosis but had otherwise completely recovered to a level comparable to that of the PBS-treated control animals. Summed histopathology scores of individual pigs are shown in Fig. 3B.

**FIG 4:**
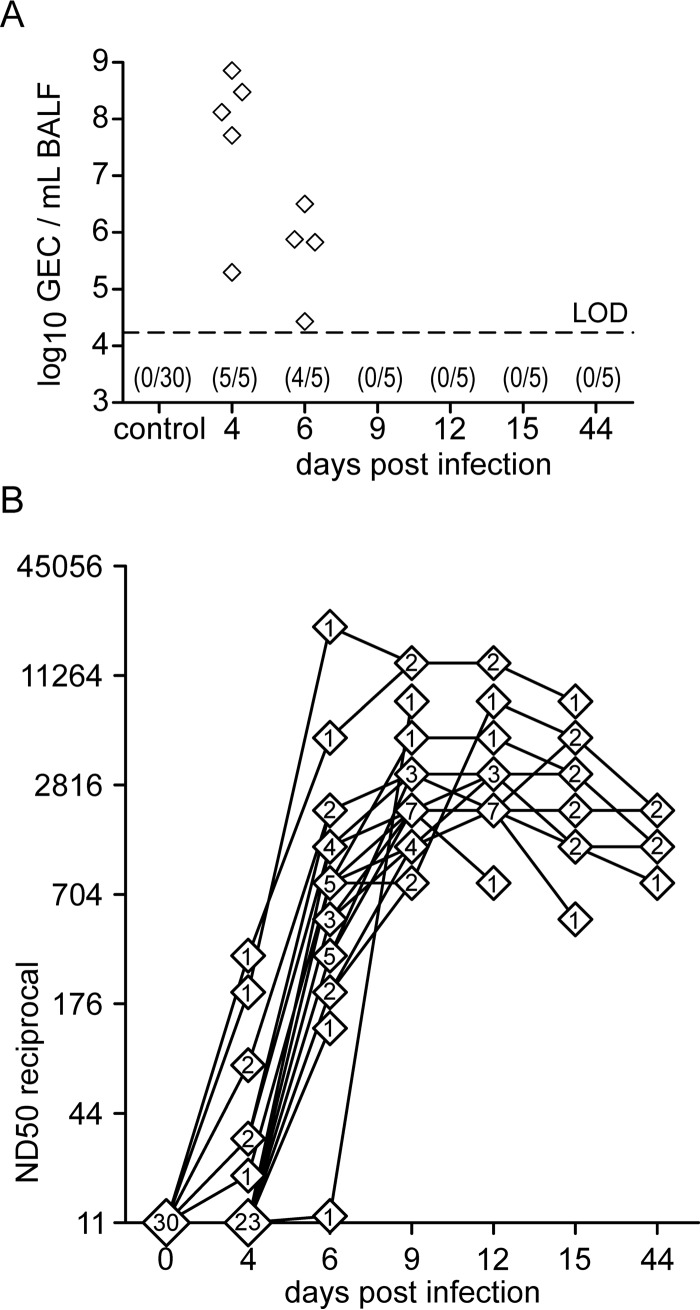
Viral load in BALF and neutralizing-antibody titer in serum. (A) Genome equivalent copies (GEC) in bronchoalveolar lavage fluid (BALF) were determined by qRT-PCR and are shown over the course of the infection and for PBS-treated control animals. The limit of detection (LOD) (17,200 GEC/ml) of the qRT-PCR assay was empirically determined using plasmid DNA and is indicated by the dashed line. Values in parentheses give the number of PCR-positive animals and the total number of animals. (B) Infection strain-neutralizing antibodies in blood were assessed by serum neutralization tests. Titers are expressed as the reciprocal 50% neutralizing dose (ND_50_). Data on 30 infected animals are shown, with lines linking values of individual animals. Numbers in diamonds indicate the number of animals with a certain reciprocal ND_50_ value at a given time point.

The viral load, as measured by qRT-PCR from BALF of euthanized animals, decreased markedly from day 4 to day 6 p.i. (Fig. 4A). In the BALF of animals euthanized from day 9 p.i. onwards, viral RNA was below the detection limit. Control animals remained negative in qRT-PCR throughout the experiment.

### Neutralizing antibodies in serum.

In sera from seven out of 30 infected animals, neutralizing antibodies against the infection strain could be detected as early as 4 days p.i. (Fig. 4B). These early responders also developed the highest antibody titers on consecutive days. By day 6 p.i., all but one of the remaining animals (24 out of 25) had detectable levels of neutralizing antibodies. In most animals, neutralizing antibodies reached their highest levels between days 9 and 15 p.i. Notably, 4 weeks later (day 44 p.i.), antibody titers were only slightly reduced in the five remaining animals. No FLUAVsw-neutralizing antibodies were detected in the sera of PBS-treated control animals (data not shown).

### *Ex vivo* Ki-67 expression and phenotype of CD4^+^ and CD8^+^ T cells.

**FIG 5:**
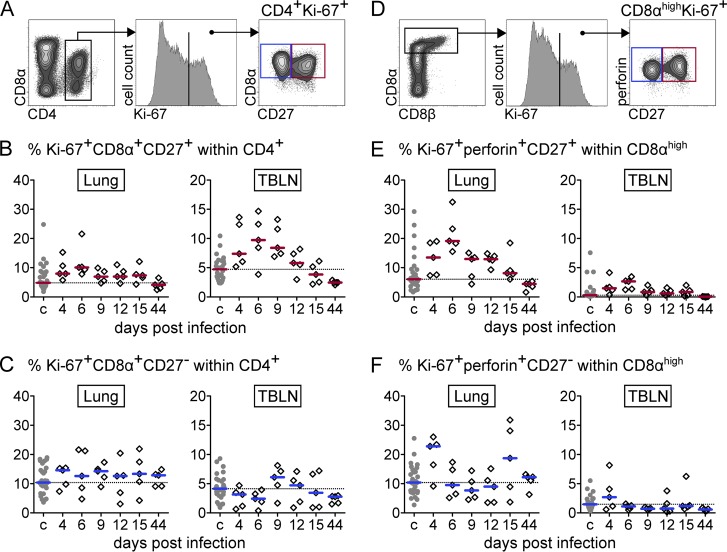
*Ex vivo* phenotypes of CD4^+^ and CD8α^high^ T cells in lungs and tracheobronchial lymph nodes (TBLN). (A) CD4^+^ T cells were gated for expression of Ki-67 and CD8α and differentiated according to their expression of CD27. (D) CD8α^high^ T cells were gated for expression of Ki-67 and perforin and differentiated according to their expression of CD27. (B, C, E, and F) Respective subsets (red gate, CD27^+^; blue gate, CD27^−^) are displayed as the percentage of total CD4^+^ T cells (B and C) or CD8α^high^ T cells (E and F) over time for infected animals (diamonds) and pooled for PBS-treated control animals (c) (gray dots). Median percent values for 30 control animals and 5 infected animals per time point are indicated by the colored bars. The dashed horizontal line indicates the level of the median of the control group.

Expression analysis of the proliferation-associated molecule Ki-67 is widely used to track and quantify the expansion phase of T cells after an experimental vaccination or infection (25, 49–51). Having previously observed Ki-67 expression kinetics reminiscent of CD8 T-cell expansion in the blood of influenza virus-infected pigs (42), we now aimed to detect an increase of recently activated T cells in lung and lung-draining lymph nodes. For this purpose, we stained freshly isolated lymphocytes for expression of Ki-67 in combination with other activation/differentiation-related molecules (CD4^+^ T cells, CD8α and CD27; CD8^+^ T cells, perforin and CD27) and analyzed them by flow cytometry (Fig. 5A and D). In pigs, the alpha chain of the CD8 molecule is expressed on activated and antigen-experienced CD4^+^ T cells (52). Porcine CD8^+^ T cells, however, express higher levels of CD8α and can be identified by a CD4^−^ CD8α^high^ CD8β^+/dim^ phenotype (53–55). For *ex vivo* analyses of CD8^+^ T cells, CD8α^high^ cells were gated (first contour plot of Fig. 5D), as weak performance of the CD8β-Zenon-Alexa 488 staining was experienced in combination with the Foxp3 staining buffer, particularly with respect to lung lymphocytes.

For CD4^+^ T cells, an increase of Ki-67^+^ CD8α^+^ CD27^+^ cells was most pronounced in the TBLN from day 4 to 9 p.i., with only a slight increase at day 6 p.i. in the lung (Fig. 5B). In contrast, for CD8^+^ T cells, the increase in cells with an early effector phenotype (Ki-67^+^ perforin^+^ CD27^+^) was most evident in the lung from day 4 to day 12 p.i., with a clear peak on day 6 p.i. (Fig. 5E). Percentages of the respective subsets lacking CD27 expression showed high pig-to-pig variation and did not reveal such a clear time-dependent pattern (Fig. 5C and F).

### IFN-γ ELISpot assay following homologous viral restimulation *in vitro*.

**FIG 6:**
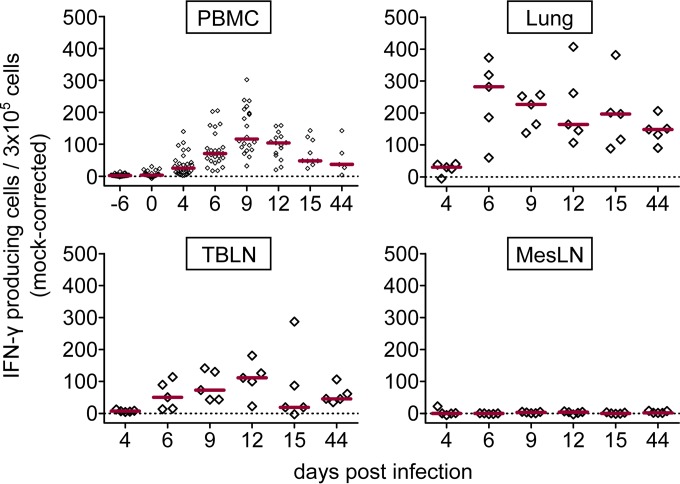
IFN-γ response to viral restimulation (ELISpot assay). Freshly isolated cells were restimulated *in vitro* with FLUAVsw (infection strain, MOI = 0.1) for 24 h or incubated with medium or mock treated. Frequencies of IFN-γ-producing cells were calculated by subtracting spot numbers of mock-incubated cultures from spot numbers detected after viral restimulation (mock corrected). Results for PBMC include surviving animals. Red bars show median values. The zero level is indicated by the dashed horizontal line.

*Ex vivo* analyses clearly pointed toward an accumulation of Ki-67^+^ T cells with an early effector phenotype at the site of infection (Fig. 5). In order to screen for a FLUAVsw-specific response within PBMC, lungs, and TBLN, we performed IFN-γ ELISpot assays upon *in vitro* restimulation (infection strain, MOI = 0.1). The first IFN-γ responses were detected within PBMC isolated on day 4 p.i. (Fig. 6). The median frequency of IFN-γ-producing PBMC peaked on day 9 p.i. and by day 15 returned to a level similar to that on day 4 p.i., where it remained until the end of the study on day 44 p.i. Within lung tissue, we observed a strong increase of IFN-γ-producing cells from day 4 to day 6 p.i. This elevated level of response in the lung more or less plateaued up to day 15 p.i. and was found to have fallen only slightly by day 44 p.i. In TBLN, FLUAVsw-specific IFN-γ responses were first detected on day 6 p.i. and were still clearly detectable on day 44 p.i. No FLUAVsw-specific IFN-γ production could be detected within cells isolated from the MesLN or within any cells isolated from PBS-treated control animals (Fig. 6 and data not shown).

### FLUAVsw-specific production of IFN-γ, TNF-α, and IL-2 by CD4^+^ and CD8^+^ T cells.

**FIG 7:**
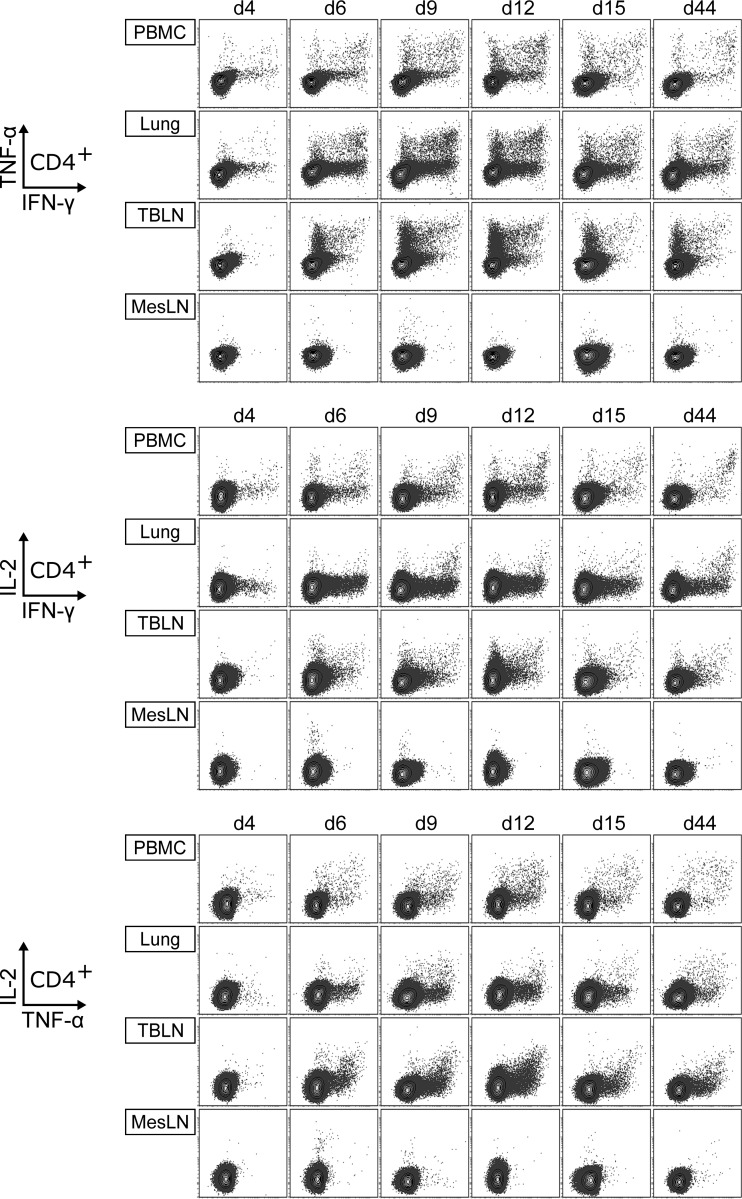
Production of IFN-γ, TNF-α, and IL-2 by CD4^+^ T cells. Intracellular cytokine staining was performed following overnight *in vitro* restimulation of freshly isolated cells with FLUAVsw (infection strain, MOI = 0.1; 18 h). Contour plots show cytokine production of CD4^+^ T cells (gated on CD4^+^ CD8β^−^ cells [not shown]) isolated from blood (PBMC), lungs, tracheobronchial lymph nodes (TBLN), and mesenteric lymph nodes (MesLN) of animals representative for each time point postinfection. Approximately 120,000 CD4^+^ T cells are shown in each contour plot.

**FIG 8:**
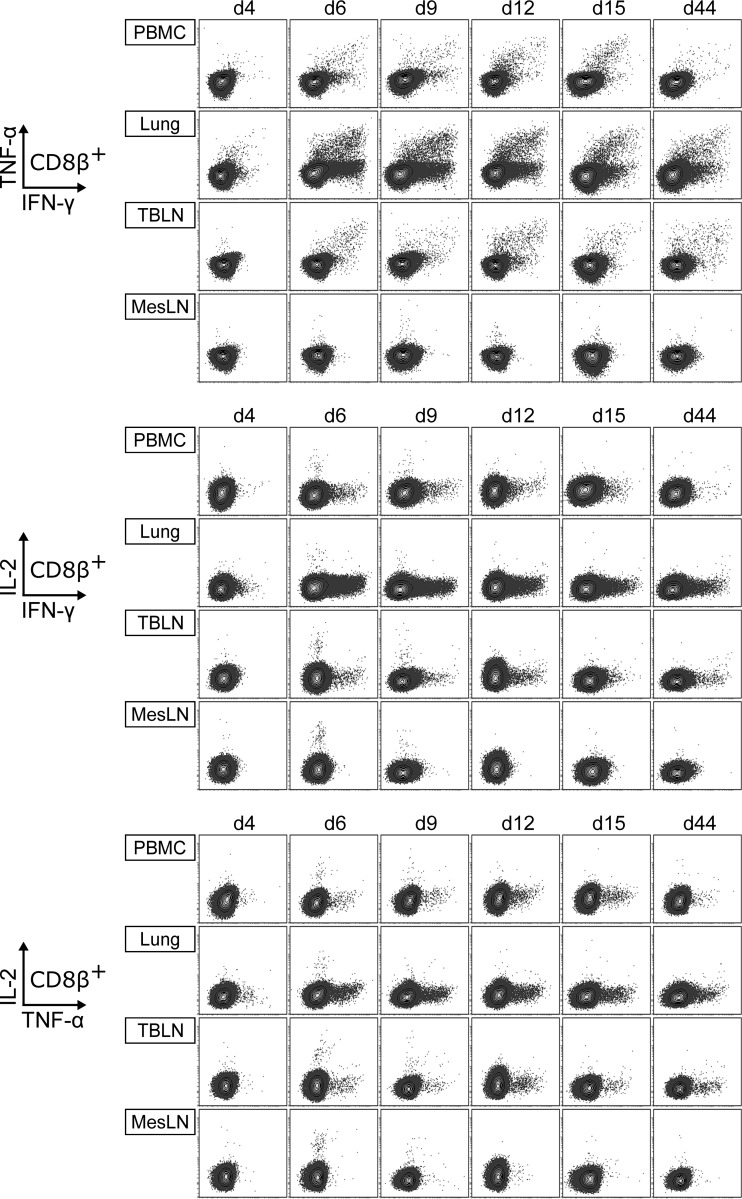
Production of IFN-γ, TNF-α, and IL-2 by CD8^+^ T cells. Intracellular cytokine staining was performed following overnight *in vitro* restimulation of freshly isolated cells with FLUAVsw (infection strain, MOI = 0.1; 18 h). Contour plots show cytokine production of CD8^+^ T cells (gated on CD4^−^ CD8β^+^ cells [not shown]) isolated from blood (PBMC), lungs, tracheobronchial lymph nodes (TBLN), and mesenteric lymph nodes (MesLN) of animals representative for each time point postinfection. Approximately 120,000 CD8^+^ T cells are shown in each contour plot.

**FIG 9:**
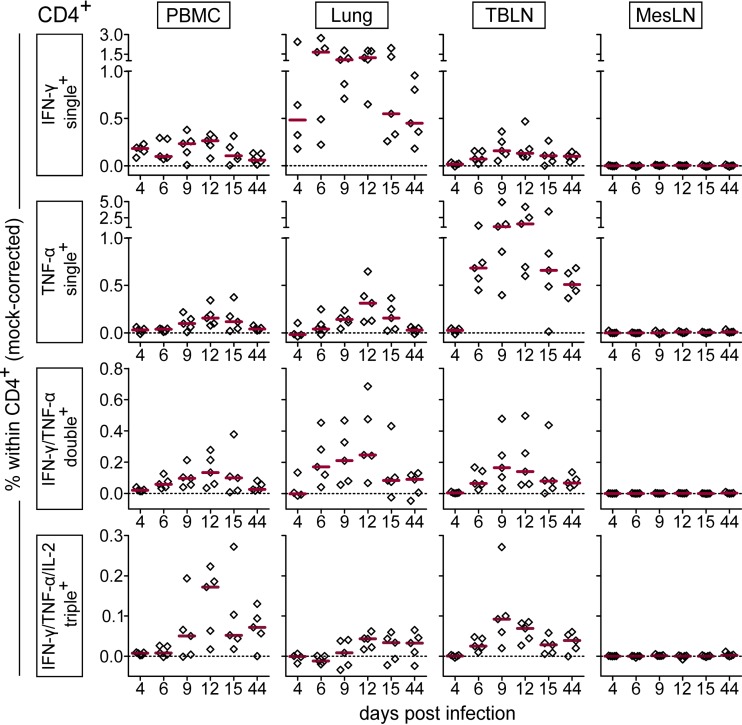
Frequency of IFN-γ-, TNF-α-, and IL-2-producing CD4^+^ T cells over the course of infection. Intracellular cytokine staining was performed following overnight *in vitro* restimulation of freshly isolated cells with FLUAVsw (infection strain, MOI = 0.1; 18 h). Mock-incubated cells served as negative controls. Frequencies of cytokine-defined subsets were obtained by Boolean gating. Mock-corrected percent values (percentage after virus restimulation minus percentage after mock incubation) of total CD4^+^ CD8β^−^ T cells are shown for the most frequent cytokine-defined subsets. The zero level is indicated by the dashed horizontal line. Red bars show the median value for 5 animals per subset, organ, and time point.

IFN-γ ELISpot assays revealed reactivity to viral restimulation in PBMC, lungs, and TBLN of FLUAVsw-infected animals. However, it should be noted that other cells (besides T cells) capable of IFN-γ production, such as NK cells, may have contributed to the IFN-γ production measured by ELISpot assays. To specifically address the phenotype and multifunctionality of responding T cells, we performed intracellular cytokine staining (ICS) for the expression of IFN-γ, TNF-α, and IL-2 following *in vitro* restimulation (infection strain, MOI = 0.1). Figures 7 and 8 show representative cytokine expression in CD4^+^ T cells and CD8^+^ T cells, respectively. Boolean gating revealed that FLUAVsw-specific IFN-γ-positive CD4^+^ T cells appeared in blood and lung as early as 4 days p.i. (Fig. 9). From day 6 or 9 p.i. onwards, singly positive TNF-α^+^ and doubly positive IFN-γ^+^ TNF-α^+^ CD4^+^ T cells could also be detected at all sites, though frequencies differed. Triply positive IFN-γ^+^ TNF-α^+^ IL-2^+^ CD4^+^ T cells could be determined in TBLN and PBMC already at day 6 p.i. but did not appear in the lung before day 9 p.i. The lung was clearly dominated by singly positive IFN-γ^+^ cells, with doubly positive IFN-γ^+^ TNF-α^+^ cells being the second most abundant subset. Singly positive TNF-α^+^ CD4^+^ T cells markedly accumulated in the TBLN. The highest frequencies of triple-cytokine-producing CD4^+^ T cells were found within PBMC, followed by TBLN, with peak frequencies detected at 9 to 12 days p.i. At the latest assessment point, i.e., 44 days p.i., FLUAVsw-specific responses comprised primarily IFN-γ single producers in the lung, TNF-α single producers in the TBLN, and IFN-γ/TNF-α/IL-2 triple producers in blood.

**FIG 10:**
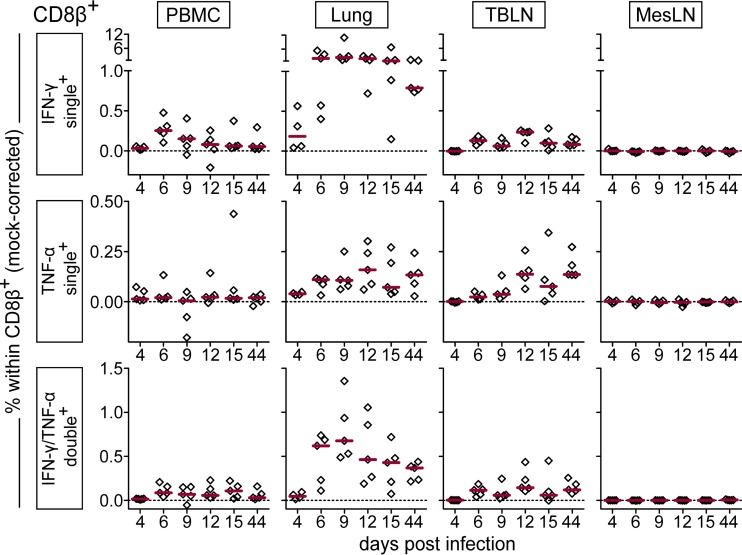
Frequency of IFN-γ- and TNF-α-producing CD8^+^ T cells over the course of infection. Intracellular cytokine staining was performed following overnight *in vitro* restimulation of freshly isolated cells with FLUAVsw (infection strain, MOI = 0.1; 18 h). Mock-incubated cells served as negative controls. Frequencies of cytokine-defined subsets were obtained by Boolean gating. Mock-corrected percent values (percentage after virus restimulation minus percentage after mock incubation) of total CD4^−^ CD8β^+^ T cells are shown for the most frequent cytokine-defined subsets. The zero level is indicated by the dashed horizontal line. Red bars show the median value for 5 animals per subset, organ, and time point.

Similar to the case for CD4^+^ T cells, the first detectable FLUAVsw-specific CD8^+^ T cells were IFN-γ single producers at day 4 p.i.; however, at this early time point, this cell subset was limited to the lung (Fig. 10). For these singly positive IFN-γ^+^ CD8^+^ T cells from the lung, a highly increased response could be detected from day 6 p.i. onwards, leading to frequencies that were up to 30 times as high as those found in PBMC or TBLN. The frequency of IFN-γ/TNF-α double producers was also highest in the lung. At 44 days p.i., around 1% and 0.5% of CD8^+^ T cells in the lung continued to respond with IFN-γ production and IFN-γ/TNF-α coproduction, respectively. In contrast to CD4^+^ T cells, CD8^+^ T cells failed to produce substantial amounts of IL-2 (Fig. 8). Both CD4^+^ and CD8^+^ T cells isolated from the mesenteric lymph nodes remained unresponsive to *in vitro* restimulation with FLUAVsw, as assessed by the lack of intracellular cytokine accumulation (Fig. 7 to 10). To confirm the influenza virus specificity of cytokine production in CD4^+^ and CD8^+^ T cells, we compared the *in vitro* response to FLUAVsw with the response to an unrelated live attenuated PRRSV strain, which is, like influenza virus, an enveloped RNA virus. Defrosted PBMC initially isolated at days 4 and 12 p.i. were restimulated in parallel with the FLUAVsw infection strain (MOI = 0.1) and the PRRSV strain (MOI = 0.2). The frequencies of IFN-γ-, TNF-α-, and/or IL-2-producing CD4^+^ and CD8^+^ T cells in PRRSV-stimulated microcultures never exceeded those frequencies found in influenza-mock-stimulated cultures (data not shown), indicating that the mock-corrected frequencies of cytokine-producing CD4^+^ and CD8^+^ T cells in Fig. 9 and 10 represent those of influenza virus-specific T cells.

### Expression of CD27 on cytokine-defined subsets of CD4^+^ and CD8^+^ T cells.

**FIG 11:**
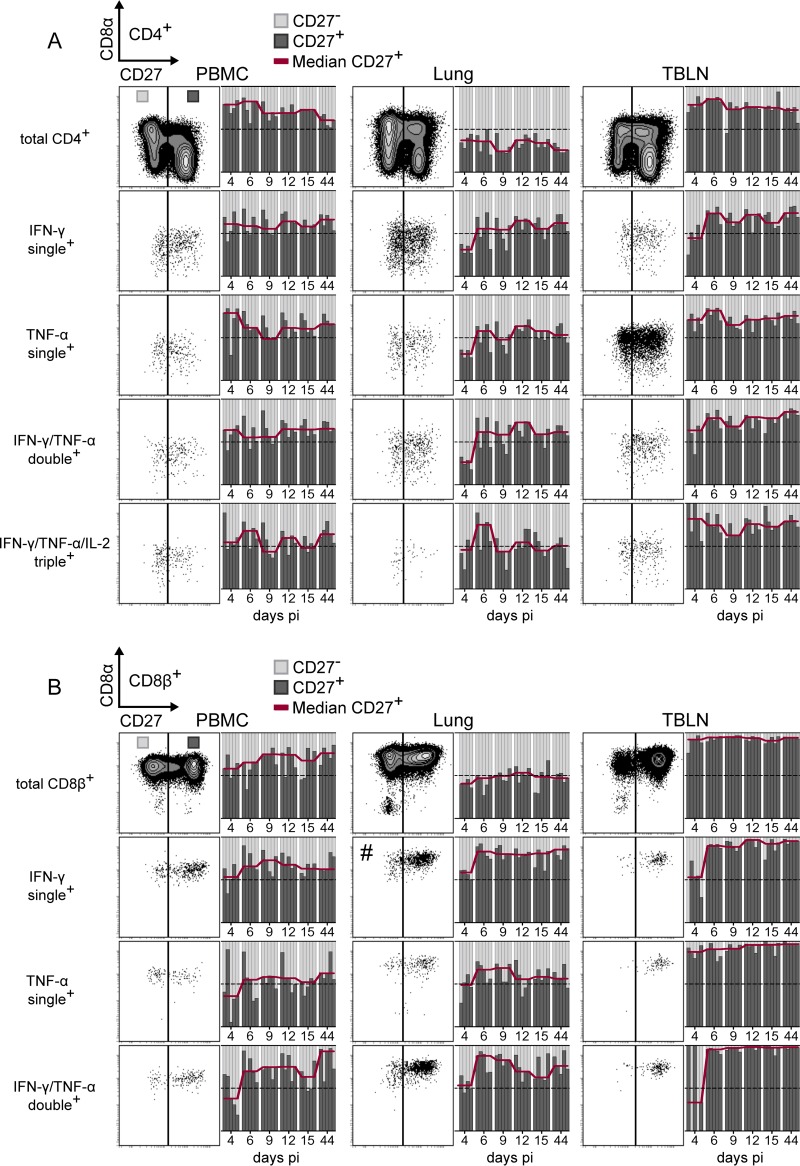
CD27 expression by cytokine-producing T cells. Intracellular cytokine staining of lymphocytes isolated from blood (PBMC), lungs, and tracheobronchial lymph nodes (TBLN) was performed after *in vitro* restimulation as described for Fig. 7 to 10. Cytokine-defined subsets identified by Boolean gating were analyzed for CD27 expression. Exemplary contour plots and dot plots show CD27 expression on total (approximately 80,000) CD4^+^ T cells (A) or CD8^+^ T cells (B) and on cytokine-defined subsets. Adjacent bar graphs show the ratio of CD27^+^ (dark gray) and CD27^−^ (light gray) cells within the respective cell population over time. Each column within a time frame corresponds to an individual animal. The red line connects median values for CD27^+^ cells of 5 animals per time point. The dotted horizontal line represents the 50% mark. For more accurate representation of subsets, the dot plot showing singly IFN-γ-producing CD8^+^ T cells in the lung (#) was set to display only 10% of events.

As CD27 has been proposed to discriminate between differentiation-related stages of porcine CD4^+^ and CD8^+^ T cells (56, 57), we looked for differences in the expression of CD27 at different time points postinfection and between different cytokine-producing T-cell subsets. As shown in the top row of Fig. 11A, total CD4^+^ T cells within PBMC and TBLN were primarily CD27^+^, whereas total CD4^+^ T cells isolated from the lung were largely negative for CD27. When looking at cytokine-producing subsets of CD4^+^ T cells, we found that in PBMC and lungs, the ratio of CD27^+^ to CD27^−^ cells was around 1:1, irrespective of the cytokines produced. For the lung this indicates that, from day 6 p.i. onwards, cytokines were preferentially produced by CD27^+^ cells.

This observation was even more pronounced for CD8^+^ T cells in the lung (Fig. 11B), where total CD8^+^ cells had a balanced 1:1 expression of CD27, and singly positive IFN-γ^+^ and doubly positive IFN-γ^+^ TNF-α^+^ cells were clearly enriched in the CD27^+^ subset. Therefore, overall FLUAVsw-specific cytokine production was dominated by T cells with an early effector phenotype or central memory phenotype across all analyzed time points.

### Cross-reactivity of CD4^+^ and CD8^+^ T cells.

**FIG 12:**
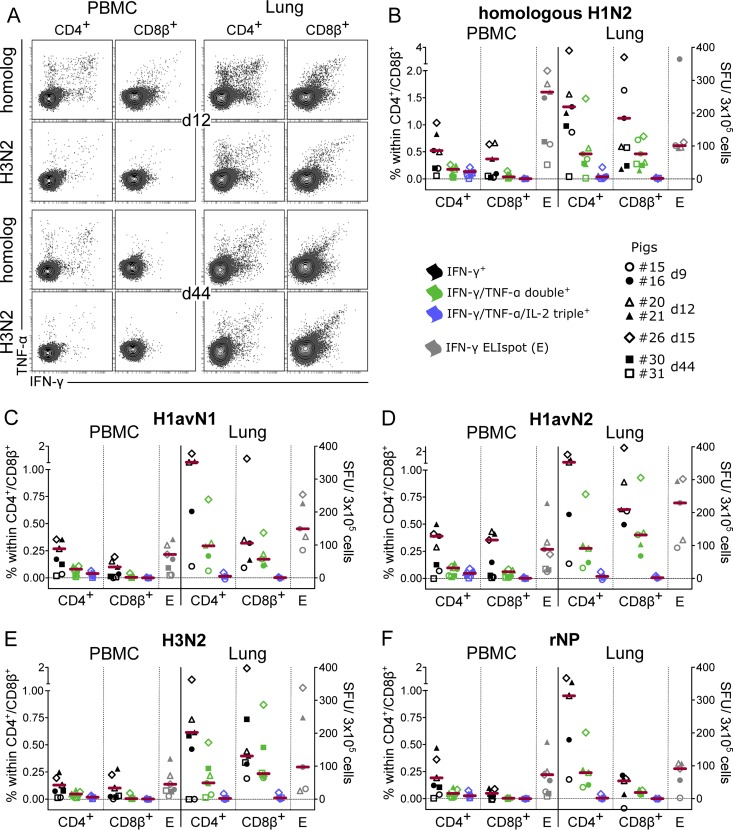
Cytokine response to restimulation with heterologous influenza A virus and nucleoprotein. For intracellular cytokine staining (ICS) and IFN-γ ELISpot assays, defrosted PBMC and lung cells were restimulated with different influenza virus strains (MOI = 0.3) and recombinant influenza virus nucleoprotein (rNP) (1 μg/ml). Mock-incubated cultures served to determine background production. (A) Contour plots show IFN-γ and TNF-α production of CD4^+^ (gated on CD4^+^ CD8β^−^ cells [not shown]) and CD8^+^ T cells (gated on CD4^−^ CD8β^+^ cells [not shown]) isolated from blood (PBMC) and lungs of pig 20 (day 12) and pig 30 (day 44). Approximately 50,000 CD4^+^ or CD8^+^ T cells are shown in each plot. (B to F) Mock-corrected frequencies of cytokine-defined subsets within CD4^+^ or CD8^+^ T cells and mock-corrected spot-forming units (SFU) (IFN-γ ELISpot assay) in response to the indicated influenza virus strains and rNP are shown for 5 to 7 animals. The zero level is indicated by the dashed horizontal line. Due to cell shortage, lung cells from day 44 were tested only by ICS after incubation with homologous H1N2, heterologous H3N2, and respective mock controls. Red bars show the median, excluding values for the last time point (day 44) p.i.

In order to test for cross-reactivity of FLUAVsw-specific T cells, defrosted PBMC and lung cells from seven infected animals, isolated from 9 days p.i. to 44 days p.i., were restimulated with heterologous FLUAVsw isolates or recombinant influenza A virus nucleoprotein (rNP), and cytokine responses were assessed by IFN-γ ELISpot assay and ICS for IFN-γ, TNF-α, and IL-2 (Fig. 12).

As shown in Table 2, the internal segments of the FLUAVsw isolate used for infection and of the three FLUAVsw isolates used for heterologous *in vitro* restimulation all belonged to the avian-derived European lineage of FLUAVsw. Analysis of HA and NA sequences assigned the isolates to three different HA and two NA subtypes/lineages. Deduced protein sequences revealed a very high degree of identity for the internal proteins of the FLUAVsw isolates (97% or higher) except for the NS-1 (Table 4). The recombinant NP derived from an H7N9 isolate had a homology of at least 94% with the NPs of all other strains.

**TABLE 4:** Percent identities of protein sequences

Protein and isolate	% identity with:				
	H1N2 IDT6142/07	H1avN1 R1738/10	H1avN2 AR1190/14	H3N2 R655/10	rNP^*a*^ (H7N9 1-BALF_RG6/13)
PB2					
IDT6142/07	100	98	98	98	
R1738/10		100	99	98	
AR1190/14			100	98	
R655/10				100	
PB1					
IDT6142/07	100	99	99	98	
R1738/10		100	98	97	
AR1190/14			100	97	
R655/10				100	
PA					
IDT6142/07	100	98	97	97	
R1738/10		100	98	98	
AR1190/14			100	98	
R655/10				100	
HA					
IDT6142/07	100	77	76	38	
R1738/10		100	88	40	
AR1190/14			100	39	
R655/10				100	
NP					
IDT6142/07	100	100	98	100	95
R1738/10		100	99	100	95
AR1190/14			100	98	94
R655/10				100	95
1-BALF_RG6/13					100
NA					
IDT6142/07	100	41	85	87	
R1738/10		100	39	41	
AR1190/14			100	88	
R655/10				100	
M1					
IDT6142/07	100	100	100	99	
R1738/10		100	100	99	
AR1190/14			100	99	
R655/10				100	
NS-1					
IDT6142/07	100	84	83	93	
R1738/10		100	92	83	
AR1190/14			100	83	
R655/10				100	

a rNP, recombinant nucleoprotein.

All heterologous virus strains (Fig. 12C to E) as well as rNP (Fig. 12F) were able to evoke cytokine production in restimulated cells. The frequency of responding cells was lower than with homologous restimulation (Fig. 12B) and was generally higher in the lung than in PBMC. When looking at the frequency of IFN-γ producers, CD4^+^ T cells were present at somewhat higher frequencies than CD8^+^ T cells, but the latter tended to contain a higher proportion of IFN-γ-/TNF-α-coproducing cells in the lung. At 44 days p.i., CD8^+^ T cells from the lung also demonstrated higher heterologous responses than their CD4^+^ counterparts (Fig. 12A and E [squares]). Cross-reactive IFN-γ-/TNF-α-/IL-2-triple-producing T cells could be identified only at low frequencies within blood-derived CD4^+^ T cells for H1avN1 and H1avN2 (Fig. 12C and D), but not for H3N2 (Fig. 12E), and were absent within CD8^+^ T cells. The latter finding is consistent with the homologous restimulations presented in Fig. 8.

The CD4 response to rNP was very similar to the response following viral restimulation. Notably, an rNP-specific IFN-γ response was also detected in CD8^+^ T cells isolated from the lungs in 4 out of 5 infected animals, although delivery of rNP-derived peptides to the major histocompatibility complex (MHC) class I presentation pathway may have been suboptimal. In summary, these findings indicate the presence of cross-reactive CD4^+^ and CD8^+^ T cells at various time points postinfection, including day 44, in particular within lung tissue but also, at low frequencies, within PBMC.

## DISCUSSION

In three controlled animal infection experiments, we analyzed the location, quality, and cross-reactivity of porcine influenza virus-responsive T cells. We were able to demonstrate that both CD4^+^ and CD8^+^ influenza virus-reactive T cells accumulate in the lungs of infected pigs from day 4 p.i. onwards, produce mainly IFN-γ and/or TNF-α, and form cross-reactive memory at the site of infection.

Following intratracheal application of virus, all 31 infected pigs developed fever and showed clinical signs of lower respiratory tract infection. Lung pathology revealed necrosis of bronchiolar epithelia until day 6 p.i., which is consistent with productive replication of influenza virus in these cells. Viral RNA in BALF fell from day 4 to day 6 p.i. and was undetectable at the remaining sampling points. Clearance of influenza virus by day 7 p.i. has been reported for influenza virus-infected swine (58), and this is consistent with the observed rapid recovery of influenza virus-infected pigs. Both neutralizing antibodies in blood and influenza virus-specific T cells in lung tissue could already be detected at low titers/frequencies at 4 days p.i. and showed a substantial increase by day 6 p.i., indicating their participation in the early control of viral infection. This early activation of T cells in a primary immune response is surprising, but NP-specific CD8^+^ T cells have been detected previously by tetramer staining or IFN-γ production in mediastinal lymph nodes and lungs of influenza virus-infected mice as early as 3 to 5 days p.i. (59, 60). Similarly, for lymphocytic choriomeningitis virus (LCMV)-infected mice, tetramer staining demonstrated the presence of LCMV NP-specific CD8^+^ T cells in the spleen from day 3 p.i. onwards (61).

Both CD4^+^ and CD8^+^ T cells isolated postinfection showed robust (multi-)cytokine responses to *in vitro* restimulation with homologous/heterologous virus and influenza virus nucleoprotein. CD8^+^ T cells produced mainly IFN-γ and/or TNF-α and lacked significant IL-2 production, confirming previous observations with blood-derived CD8^+^ T cells from influenza virus-infected pigs (42) and nonhuman primates (62). In humans, preexisting IFN-γ^+^ CD8^+^ T cells lacking IL-2 production, and not IFN-γ^+^ IL-2^+^ CD8^+^ T cells, could be correlated with decreased symptom scores upon natural infection with pandemic H1N1 influenza virus (26), arguing against the necessity of CD8-derived IL-2 for clinical protection. Indeed, IFN-γ is often described as a key antiviral cytokine. Mouse studies have demonstrated the importance of rapid IFN-γ production by airway-resident memory CD8^+^ T cells for protection against heterologous influenza virus challenge (22). In addition, CD4-derived IFN-γ was shown to be necessary for the generation of lung-resident CD8 T-cell memory (63). Thus, the dominant populations of FLUAVsw-specific IFN-γ-producing CD4^+^ and CD8^+^ T cells identified in various organs in this study are likely to have contributed to controlling the infection.

Multifunctionality, i.e., the simultaneous production of more than one cytokine or effector molecule at the single-cell level, has been proposed as an important quality measure of T-cell responses (64, 65). Perforin is one of the main effector molecules of CD8^+^ T cells, mediating apoptosis of influenza virus-infected cells and viral clearance (16). Evidence that multifunctionality of CD8^+^ T cells may translate into increased cytotoxic activity has been provided by Lichterfeld et al. (66), who found increased perforin expression and stronger *ex vivo* cytotoxic activity of CD8^+^ T cells producing both IFN-γ and TNF-α than CD8^+^ T cells producing IFN-γ only. In the present study, we were able to detect a considerable proportion of doubly IFN-γ/TNF-α-producing CD4^+^ and CD8^+^ T cells in porcine lungs infected with FLUAVsw. Moreover, we could detect peak frequencies of proliferating (Ki-67^+^) and perforin-expressing CD8^+^ T cells in lungs at 6 days p.i., clearly suggesting that CD8^+^ T cells play a role in viral clearance by cytolytic activity.

In order to distinguish between differentiation stages of influenza virus-responsive T cells, we coanalyzed CD27 expression. Porcine CD27 was shown to be differentially expressed on antigen-experienced (CD8α^+^) CD4^+^ T cells, and functional analyses suggested a division into central memory (CD27^+^) and effector memory (CD27^−^) cells (56). For perforin-expressing CD8^+^ T cells in pigs, age-related changes suggested a division into early (CD27^+^) and late (CD27^−^) effector cells (57). In mice, engagement of costimulatory CD27 has been shown to promote effector expansion as well as survival and memory transition of T cells (67–69). Ballesteros-Tato et al. (59) correlated CD27 expression with the proliferation of effector CD8 T cells by showing that the *in vivo* expansion of influenza virus nucleoprotein-specific CD8^+^ T cells was dependent on CD70-CD27 interaction. Our *ex vivo* observations support this finding and extend it to include CD4^+^ T cells, as kinetics reminiscent of effector expansion were evident only in Ki-67^+^ T cells expressing CD27. We also addressed the expression of CD27 on influenza virus-specific T cells over the course of the infection and with respect to the cytokines produced. In the lung, we found that cytokine-producing influenza virus-specific T cells were clearly skewed toward CD27 expression. This propensity for CD27 expression was most pronounced for IFN-γ-producing CD8^+^ T cells and may be related to better survival and memory transition, supported by the high frequencies of IFN-γ-producing CD8^+^ T cells both in the acute phase and 5 weeks after clearance of infection.

Indeed, the high numbers of FLUAVsw-specific T cells in lungs, long after clearance of infection, strongly suggest the formation of lung-resident T-cell memory, which has been shown to mediate superior protection against influenza virus challenge in mice (19–22). We also detected considerable memory responses in lung-draining lymph nodes and peripheral blood, possibly indicating immunological memory that can be rapidly recruited to the lung to support frontline memory T cells upon secondary infection (70).

In contrast to most neutralizing antibodies, T cells have the potential to be broadly reactive across influenza virus subtypes (14). T cells recognize internal epitopes that, due to functional constraints (71), are less likely to accumulate nucleotide substitutions and are therefore highly conserved among different influenza virus strains. Although cross-reactive T-cell memory cannot confer sterilizing immunity, their presence has been shown to be associated with reduced disease severity in humans (25–27). For pigs, reduced viral replication and shedding have previously been shown in consecutive experimental infections with different influenza A virus strains (36, 72–75). One study demonstrated that this was associated with an increase of CD8^+^ T cells in lung tissue (33). Moreover, by infecting pigs with a genetically modified influenza virus strain that suppresses the HA signal sequence (S-FLU) (76) and that does not induce neutralizing antibodies, Morgan et al. (41) found a correlation between IFN-γ-producing CD8^+^ and CD4^+^ T cells in BALF and a reduction of viral load in nasal swabs and lungs upon homologous and heterologous challenge. In the present study, we demonstrated T-cell reactivity against three heterologous influenza virus strains and nucleoprotein. Influenza virus nucleoprotein evoked CD4 T-cell responses comparable to those of heterologous virus, in both magnitude and quality, suggesting an immunodominant role of nucleoprotein for porcine CD4^+^ T cells.

Overall, our data support the idea that in the pig, a natural influenza virus host, T cells are actively involved both in the clearance of primary infection and in the control of heterologous reinfection. We are confident that this and ensuing work on the porcine T-cell response to influenza virus will strengthen the position of pigs as large-animal models for human influenza virus infection and will immediately aid the development of more effective vaccines for swine. Improved control of influenza virus replication in pigs will reduce the chances of influenza virus reassortments, as well as reducing pig-human transmission, thereby lowering the risk of swine-mediated pandemic outbreaks.
